# Molecular detection of zoonotic filarioids in *Culex* spp. from Portugal

**DOI:** 10.1111/mve.12524

**Published:** 2021-05-04

**Authors:** R. R. S. Manoj, M. S. Latrofa, M. A. Cavalera, J. A. Mendoza‐Roldan, C. Maia, D. Otranto

**Affiliations:** ^1^ Department of Veterinary Medicine University of Bari Valenzano Italy; ^2^ Global Health and Tropical Medicine (GHTM), Instituto de Higiene e Medicina Tropical (IHMT) Universidade NOVA de Lisboa, Rua da Junqueira, 100 Lisbon, 1349‐008 Portugal; ^3^ Faculty of Veterinary Sciences Bu‐Ali Sina University Hamedan Iran

**Keywords:** *Acanthocheilonema reconditum*, *Culex* spp., *Dirofilaria immitis*, *Dirofilaria repens*, *Onchocerca lupi*, Portugal, *Wolbachia*

## Abstract

To investigate the role of dipterans in the transmission of *Onchocerca lupi* and other zoonotic filarioids, samples were collected from different sites in Algarve, southern Portugal, morphologically identified and molecularly tested for filarioids. *Culex* sp. (72.8%) represented the predominant genus followed by *Culicoides* sp. (11.8%), *Ochlerotatus* sp. (9.7%), *Culiseta* sp. (4.5%), *Aedes* sp. (0.9%) and *Anopheles* sp. (0.3%). Nineteen (2.8%) specimens scored positive for filarioids, with *Culex pipiens quinquefasciatus* (2%) positive for *Dirofilaria immitis* (1.4%), *Dirofilaria repens*, *Acanthocheilonema reconditum*, *Onchocerca lupi*, unidentified species of Filarioidea (0.2%, each) and Onchocercidae (0.6%). Additionally, *Culiseta longiareolata* (6.5%), *Ochlerotatus caspius* (3%) and *Culex laticinctus* (0.2%) scored positive for unidentified Onchocercidae, *A. reconditum* and for *O. lupi*, respectively. This is the first report of the occurrence of DNA of *O. lupi*, *D. repens* and *A. reconditum* in *Culex* spp. in Portugal. Information regarding the vectors and the pathogens they transmit may help to adopt proper prophylactic and control measures.

## Graphical abstract

First report of the occurrence of the DNA of *Onchocerca lupi*, *Dirofilaria repens* and *Acanthocheilonema reconditum* in *Culex* spp. from Portugal.
Unidentified species of Filarioidea and Onchocercidae were detected in mosquitoes from Portugal.
The detection of *Wolbachia* supergroup E in *Culex laticinctus* positive for *O. lupi* is new to science and deserves further investigation.

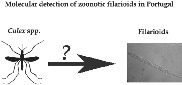

## Introduction

Arthropods include important vectors of pathogens, being mainly distributed and abundant in tropical and temperate regions (Schaffner *et al*., 2013). Among them, mosquito species belonging to several genera (e.g., *Anopheles*, *Culex*, *Aedes*, *Ochlerotatus* and *Mansonia*) are recognized as vectors of viruses, bacteria and filarioids causing diseases in many animal species, including humans (Otranto *et al*., 2013a). Similarly, biting midges (*Culicoides* spp.) are recognized as vectors of other filarioids (i.e., *Onchocerca cervicalis*, Railliet and Henry, 1910 and *Onchocerca gutturosa*, Neumann, 1910 of horses and cattle, respectively) (Takaoka *et al*., 1995). *Dirofilaria immitis* (Leidy, 1856), the causative agent of canine heartworm disease, is distributed in tropical and temperate regions (Otranto *et al*., 2013a), whereas *Dirofilaria repens* (Railliet and Henry, 1911), causing canine subcutaneous dirofilariosis, is present in continental and eastern European countries (Capelli *et al*., 2018). The majority of human cases of infection by *Dirofilaria* spp. are associated with *D. repens* in Europe and *D. immitis* in the Americas (Dantas‐Torres & Otranto, 2013). In addition, an increasing number of human infections caused by these filarioids have been reported in European countries (Diaz, 2015). Less studied filarioids such as *Acanthocheilonema reconditum* (Grassi, 1890) and *Cercopithifilaria* spp. are showing an increase in prevalence (up to 15.9%) among canine populations in Europe (Otranto *et al*., 2012). Furthermore, *Onchocerca lupi* (Rodonaja, 1967) has gained the interest of the scientific community as a zoonotic agent both in the USA, Europe, northern Africa and Middle East Asia (Otranto *et al*., 2015; Colella *et al*., 2018). Indeed, after the first case report of human ocular onchocercosis caused by *O. lupi* in Turkey (Otranto *et al*., 2011), up to 19 patients have been diagnosed positive for this parasite, worldwide (i.e., Germany, Tunisia, Hungary, Greece, Turkey, Iran and U.S.A.) (Berry *et al*., 2014; Dudley *et al*., 2015; Grácio *et al*., 2015). Though *O. lupi* has been previously detected in a wolf in Republic of Georgia many decades ago (Rodonaja, 1967), data on the infection in dogs and cats are limited to a few case reports from southern (Greece, Portugal, Spain) and central Europe (Germany, Hungary, Switzerland) (Otranto *et al*., 2013b; Grácio *et al*., 2015; Maia *et al*., 2015; Miró *et al*., 2016). In particular, the southern area of Portugal, Algarve region, has been spotted as one of the major foci of infection for *O. lupi* in canine populations and, after the first case report in 2010 (Faísca *et al*., 2010), up to 8.3% of infection was detected in apparently healthy dogs (Otranto *et al*., 2013b). Despite these prevalence data, information on the epidemiology and biological cycle of *O. lupi* is still minimal or lacking. Indeed, though there are reports of *O. lupi* putative vector (e.g., *Simulium tribulatum*, Lugger, 1897 in the U.S.A.) (Hassan *et al*., 2015), the final confirmation has not been achieved. Moreover, evidences suggest that *D. repens* has spread faster than *D. immitis* from the endemic areas of southern Europe to North, while the prevalence of seropositive humans is increasing in western and eastern Europe (Capelli *et al*., 2018; Fontes‐Sousa *et al*., 2019). In particular, *D. immitis* was serologically detected in humans and dogs throughout Portugal (Alho *et al*., 2018; Fontes‐Sousa *et al*., 2019), as well as, *D. repens* in one dog (Maia *et al*., 2016) from Algarve region, suggesting that *Dirofilaria* spp. are endemic in this country.

The current study aimed to identify the mosquito and biting midge populations present in specific sites of Algarve region and to molecularly detect filarioids they carry, in order to gain more information about their vector role in areas where *O. lupi* and *D. immitis* occur in sympatry.

## Material and methods

### Sample collection

**Fig. 1:**
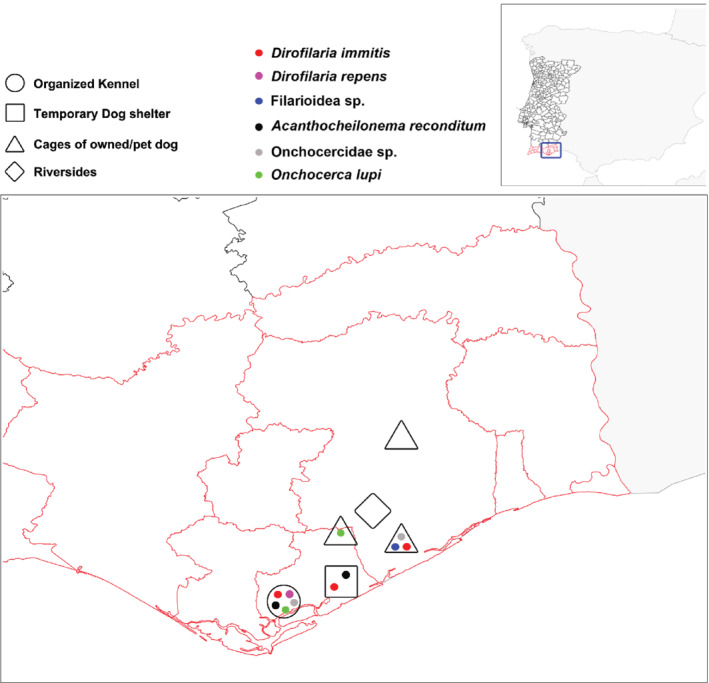
Map of South‐East Algarve region, Portugal. Sampling sites and filarioids detected are indicated.

From June to August 2018, flies were collected from four different collection sites of Algarve region (southern Portugal), using CO_2_‐baited CDC light and BG‐sentinel‐2 mosquito traps baited with dry ice and BG lure containing ammonia, lactic acid and caproic acid (Biogents, Regensburg, Germany). Sampling areas (Fig. 1) were selected primarily based on previous history of dogs infected by *O. lupi* (Otranto *et al*., 2013b) in an organized kennel, in and around the cages of privately owned dogs (i.e., n = 3), in a temporary dog shelter, and in the riversides and near areas frequented by hunting dogs. Sampling was carried out throughout the day at weekly intervals in all six sites and the collected samples were kept at −4 °C until transferred to the laboratory. Female mosquitoes and *Culicoides* were identified up to genus level based on morphological keys of Ribeiro & Ramos (1999) and Mathieu *et al*. (2012), respectively, dissected under a stereomicroscope to find the larval stages of filarioids, and placed in single tubes containing 70% ethanol until further molecular processing.

### DNA isolation, molecular and sequencing analyses

Genomic DNA was isolated from individual female flies using GenUP gDNA Kit (Biotechrabbit, Germany), following the manufacturer's instructions. All samples were tested molecularly for the DNA of filarioids by conventional PCR (cPCR) using two set of primers targeting partial 12S rRNA and cytochrome *c* oxidase subunit 1 (*cox*1) genes, respectively. Each fly that tested positive for filarioid was molecularly identified using primers targeting partial *cox*1 gene. The source of blood meal was analysed in positive flies using the primer pairs targeting *cytochrome b* gene and cPCR run protocol previously described (Appendix S1). Positive dipterans were also tested for the endosymbiont, *Wolbachia pipientis* infection using 16S rRNA and *Wolbachia* surface protein (*wsp*) genes. All amplified PCR products were visualised in 2% agarose gel containing Gel Red® nucleic acid gel stain (VWR International PBI, Milan, Italy) and documented in Gel Logic 100 (Kodak, New York, NY, U.S.A.). The PCR products were purified and sequenced in both directions using the same primers, employing the Big Dye Terminator v.3.1 chemistry in a 3130 Genetic analyser (Applied Biosystems, Foster City, CA, U.S.A.) in an automated sequencer (ABI‐PRISM 377). Nucleotide (nt) sequences were edited, aligned and analysed using BioEdit and compared with available sequences in the GenBank using Basic Local Alignment Search Tool (BLAST; http://blast.ncbi.nlm.nih.gov/Blast.cgi). All specimens were also screened for the DNA of *O*. *lupi* using quantitative real‐time PCR (qPCR) assay and run protocol described elsewhere. The details of the primers used in the current study are given in supplementary file (Appendix S1).

### Phylogenetic analysis

Representative sequences of the 12S rRNA and *cox*1 genes of filarioids and *wsp* gene of *W. pipientis* were included along with the sequences available in the GenBank database for phylogenetic analyses. Phylogenetic relationships were inferred using Maximum Likelihood (ML) method based on General Time Reversible model (Nei & Kumar, 2000) with discrete Gamma distribution (+*G + I*) to model evolutionary rate differences among sites for *cox*1, Hasegawa‐Kishino‐Yano and Tamura 3‐parameter models with +*G* for 12S rRNA and for *wsp* genes, respectively (Hasegawa *et al*., 1985; Tamura, 1992; Kumar *et al*., 2018), selected by best‐fit model (Nei & Kumar, 2000). Evolutionary analyses were conducted on 1000 bootstrap replications using the MEGA X software (Tamura *et al*., 2013). Homologous sequences from *Filaria martis* and *Thelazia callipaeda* were used as outgroups (*cox*1: AM042552, AJ544880; 12SrRNA: AJ544858).

### Statistical analysis

Prevalence of filarioid infection among flies (proportion of insects infected by larval stages of different filarioid species on the total population of insects collected from different location of Portugal) was assessed. Statistical analysis was done using StatLib software. Exact binomial 95% confidence intervals (CI) were established for proportions.

## Results

The most prevalent species of dipteran collected belonged to the genus *Culex* (n = 501, 72.8%), followed by *Culicoides* (n = 81, 11.8%), *Ochlerotatus* (n = 67, 9.7%), *Culiseta* (n = 31, 4.5%), *Aedes* (n = 6, 0.9%) and *Anopheles* (n = 2, 0.3%). Of the 688 specimens collected, the highest number was from cages of privately owned dogs (45.5%) (Table 1), whereas the highest prevalence of filarioid infection (3.2%) was recorded in mosquitoes collected from organised kennel (Table 2). Only one mosquito scored positive by a larva of *D. immitis* after dissection. Nineteen mosquito specimens (2.8%; 95% CI: 1.7–4.3) scored molecularly positive for filarioids, without coinfections and with *Culex* spp. as the most prevalent infected genus (2.2%; 95% CI: 1.3–3.6). At the molecular detection, 14 specimens of *Culex pipiens quinquefasciatus* (Say, 1823; 100% nt identity with MK575480) scored positive for *D. immitis* (n = 7, 1.4%; 95% CI: 0.6–2.9), Onchocercidae (n = 3, 0.6%; 95% CI: 0.2–1.8), *O. lupi*, *D. repens*, *A. reconditum* and Filarioidea (n = 1, 0.2%, each; 95% CI: 0.02–1.1) by cPCR (Table 2). Moreover, *Culex laticinctus* (Edwards, 1913; 95.01% nt identity with MT993489), *Ochlerotatus caspius* (Pallas, 1771; 99.62% nt identity with MT993477) and *Culiseta longiareolata* (Macquart, 1838; 100% nt identity with MT993479) scored positive for *O. lupi* (n = 1, 0.2%; 95% CI: 0.02–1.1), *A. reconditum* (n = 2, 3%; 95% CI: 0.5–10.2) or for Onchocercidae (n = 2, 6.5%; 95% CI: 1.2–20.7), respectively (Table 2). None of the *Anopheles* spp. (n = 2), *Aedes* spp. (n = 6) and *Culicoides* spp. (n = 81) scored positive (data not shown). *Wolbachia pipientis* (Hertig, 1936) was only detected in 15 filarioid‐infected *Culex* spp.. Detection by qPCR confirmed the positivity of *Cx. p. quinquefasciatus* and of *Cx. laticinctus* for *O. lupi*. Of the 19 positive mosquitoes, 6 scored positive for the host blood meal, with two *Oc. caspius* positive for dog and human, two *Cs. longiareolata* for avian and two *Cx. p. quinquefasciatus* for human (Appendix S2). Blast analyses of all sequences of *D. immitis*, *D. repens*, *O. lupi* and *A. reconditum* displayed a nt identity of 100% with those available in GenBank database for both genes examined (12SrRNA: KF707482, KX265091, KC686903, MT252013; *cox*1: MN945948, MT012806, KX853327; JF461456). An identity ranging from 96.3 to 98.3% was obtained for 12S rRNA sequences of Onchocercidae and Filarioidea (KR676614, JX870434) and from 98.9 to 99.1% for *cox*1 sequences of Filarioidea (LC107819). All *Culicoides* spp. scored negative for filarioids. The *wsp* sequences of *W. pipientis* detected in *Cx. p. quinquefasciatus* showed a nt identity from 99.5% to 100% with that available from GenBank (MH218812) with the exclusion of that found in *Cx*. *laticinctus*, which showed a nt identity of 99.3% with that of *Culex decens* (Theobald, 1901; MK033274).

**Table 1:** Number and percentage of genera of dipterans collected from Algarve region, Portugal, divided according to the genus and sampling site of collection.

Description of sampling sites	*Culex* Tot (%)	*Ochlerotatus* Tot (%)	*Aedes* Tot (%)	*Culiseta* Tot (%)	*Anopheles* Tot (%)	*Culicoides* Tot (%)	Total (%)
Organized kennel (n = 1)	270 (95.4)	9 (3.2)	3 (1.1)	1 (0.4)	—	—	283 (41.1)
Temporary dog shelter (n = 1)	14 (18.2)	55 (71.4)	2 (2.6)	1 (1.3)	—	5 (6.5)	77 (11.2)
Cages of owned/pet dog (n = 3)	203 (64.9)	2 (0.6)	1 (0.3)	29 (9.3)	2 (0.6)	76 (24.3)	313 (45.5)
Riversides (n = 1)	14 (93.3)	1 (6.7)	—	—	—	—	15 (2.2)
Total	501 (72.8)	67 (9.7)	6 (0.9)	31 (4.5)	2 (0.3)	81 (11.8)	688

**Table 2:** Number (positive/total) and prevalence (%) of filarioids in dipterans divided according to sampling sites from Algarve region, Portugal.

	*Culex* (n = 501)						*Ochlerotatus* (n = 67)	*Culiseta* (n = 31)
Sampling site	*D. immitis*	*D. repens*	*A. reconditum*	*O. lupi*	*Onchocercidae* sp.	*Filaroidea* sp.	*A. reconditum*	*Onchocercidae* sp.
Organized kennel (n = 1)	4/270 (1.5)	1/270 (0.4)	1/270 (0.4)	1/270 (0.4)	1/270 (0.4)	0/270	1^†^/9 (11.1)	0/1
Temporary dog shelter (n = 1)	1/14 (7.1)	0/14	0/14	0/14	0/14	0/14	1^†^/55 (1.8)	0/1
Cages of owned/pet dog (n = 3)	2/203 (1.0)	0/203	0/203	1*/203 (0.5)	2/203 (1.0)	1/203 (0.5)	0/2	2^‡^/29 (6.9)
Riversides (n = 1)	0/14	0/14	0/14	0/14	0/14	0/14	0/1	—

* *Culex laticinctus*.

^†^ *Ocharotatus caspius*.

^‡^ *Culiseta longiareolata*; Positive specimens of *Culex* without asterisk belongs to *Culex pipiens quinquiefasciatus*. None of the *Anopheles* sp. (n = 2), *Aedes* sp. (n = 6) *Culicoides* sp. (n = 81) scored positive.

Mosquitoes molecularly identified were indicated with the following symbols.

**Fig. 2:**
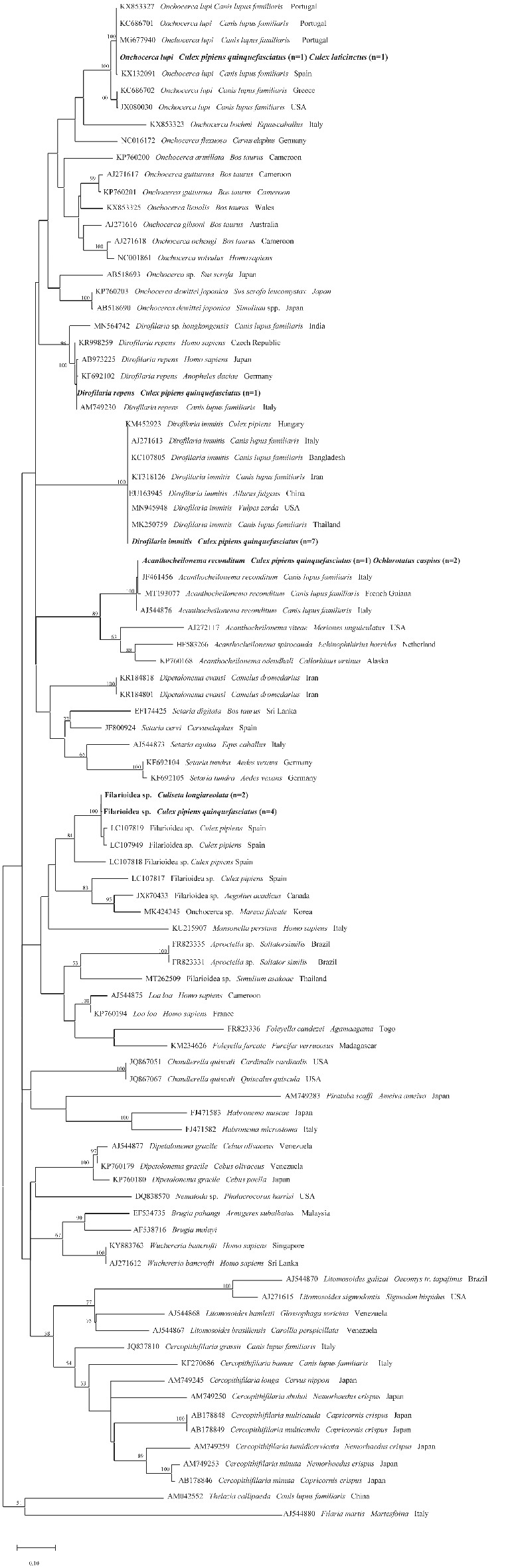
Phylogenetic relationship of filarioids detected in this study (in bold) and other filarioids available from GenBank based on cytochrome *c* oxidase sequences. Evolutionary analysis was conducted on 1000 bootstrap replications using Maximum Likelihood method and General Time Reversible model. *Filaria martis* was used as outgroup. GenBank accession number, host species and country of origin are indicated.

**Fig. 3:**
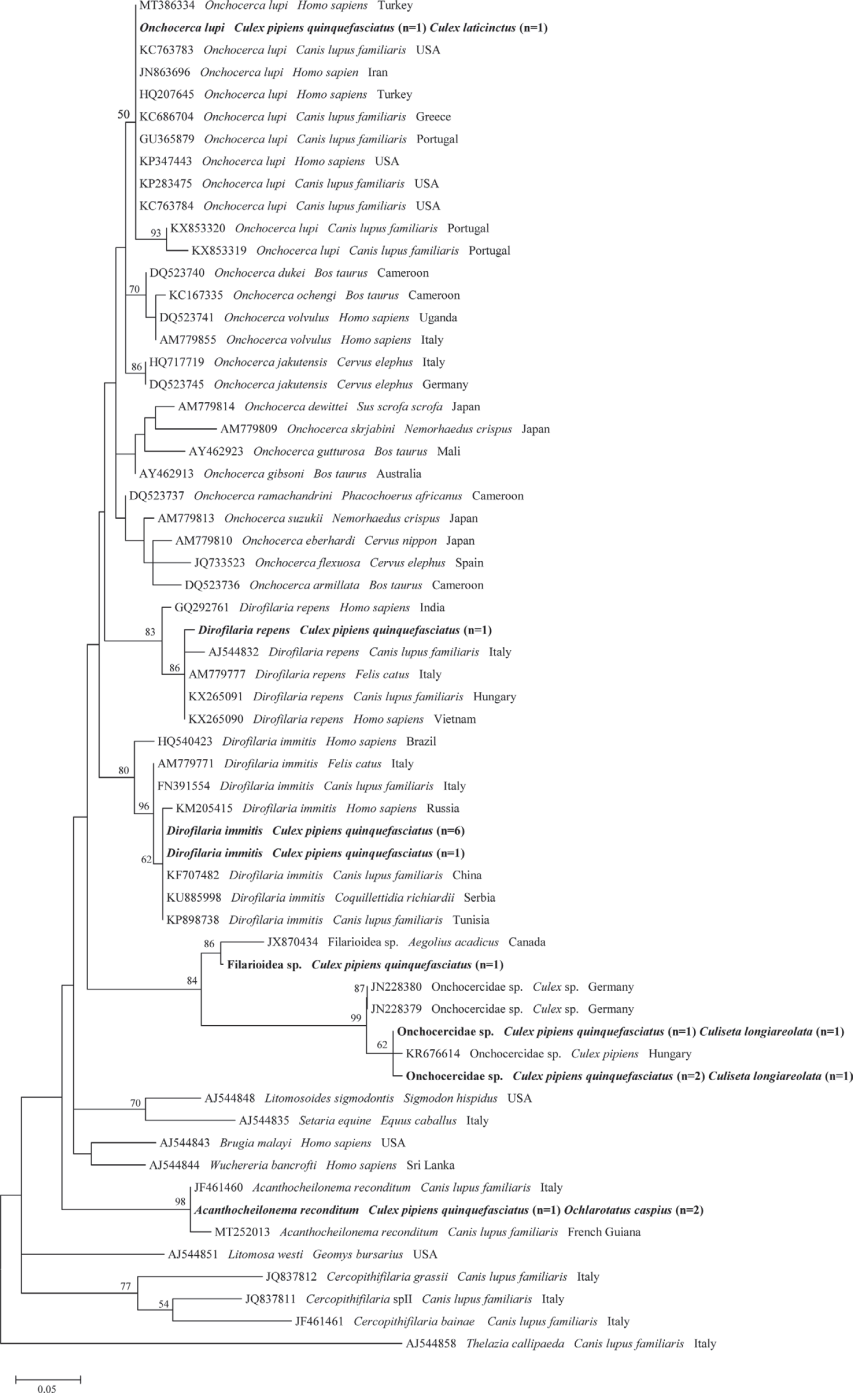
Phylogenetic relationship of filarioids detected in this study (in bold) and other filarioids available from GenBank based on a partial sequence of the 12S rRNA gene. Evolutionary analysis was conducted on 1000 bootstrap replications using Maximum Likelihood method and Hasegawa‐Kishino‐Yano model. *Thelazia callipaeda* was used as outgroup. GenBank accession number, host species and country of origin are indicated.

Phylogenetic analyses support molecular identification by clustering all the representative sequences of *D. immitis*, *D. repens*, *O. lupi and A. reconditum* in the corresponding species clades supported by high bootstrap value for *cox*1 and 12S rRNA trees (Figs 2 and 3). In particular, both 12S rRNA and of *cox*1 phylogenetic dendrograms clustered sequences of unidentified Onchocercidae and Filarioidea within the clades of the same species, respectively, and those of *O. lupi* within a species‐specific clade which includes reference sequences from Portugal and Spain, and as a sister clade which includes sequences of *O. lupi*, with the exclusion of other *Onchocerca* species (Figs 2 and 3).

**Fig. 4:**
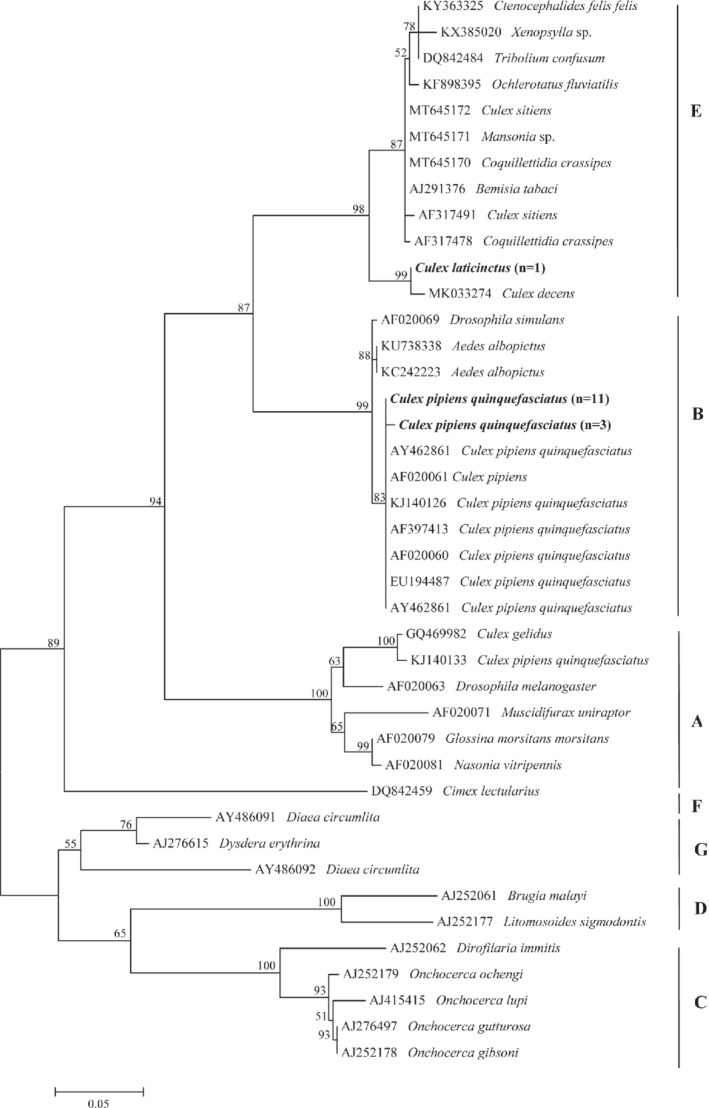
Phylogenetic relationship of *Wolbachia* detected in this study (in bold) and other available from GenBank belonging to different supergroups based on a partial sequence of the *Wolbachia surface protein* (*wsp*). Evolutionary analysis was conducted on 1000 bootstrap replications using Maximum Likelihood method and Tamura 3‐parameter model. GenBank accession number and host species are indicated.

Similarly, the ML analyses of *Wolbachia* sequences yielded strict consensus in overlapping the topology of trees obtained from *wsp* (Fig. 4) and 16S rRNA (data not shown) genes, with a strong bootstrap value (up to 99%) for each clade (Fig. 4). All representative sequences of *Wolbachia* detected in *Cx. p. quinquefasciatus* clustered in the same clade of the reference *Wolbachia* sequences that belong to supergroup B, excluding the monophyletic clade which grouped the sequences found in *Cx*. *laticinctus* with those of the reference supergroup E (Fig. 4). Representative sequences of all filarioids and *Wolbachia* were deposited in GenBank (Accession numbers: MW242746–48, MW243588, MW254895‐901, MW435608‐14; Appendix S3).

## Discussion

This study assessed the presence of filarioids in dipterans collected from specific sites in Algarve region, southern Portugal. In particular, DNA of *O. lupi* was detected for the first time in mosquito specimens belonging to *Culex* genus, as well as that of *D. repens* in the same mosquito genus, in this country. The predominance of *Culex* spp. (72.8%) herein collected was supported by previous data where a high prevalence of mosquito species belonging to this genus (i.e., *Cx. pipiens*, 52% and *Culex theileri*, Theobald, 1903, 29%) was reported (Freitas *et al*., 2012). The finding of different dipteran genera, such as *Culex*, *Ochlerotatus*, *Aedes*, *Culiseta*, *Anopheles* and *Culicoides*, may be due to the favourable climatic conditions recorded in the Algarve province. For example, variation in relative abundance and species diversity (i.e., *Oc. caspius Cx. pipiens* and *Anopheles atroparvus*, Van Thiel, 1927) has been observed from season to season and every year in the above mentioned region (Freitas *et al*., 2012; Osório *et al*., 2014; Silva *et al*., 2019). Indeed, the presence of wetlands and lagoons, used by migratory birds, can favour the abundance and diversity in mosquito species populations, which may perpetuate the biological cycle of pathogens and/or potentially transmit them (Freitas *et al*., 2012).

The most prevalent filarioid detected in the current study was *D. immits* (1%) found in *Cx. p. quinquefasciatus*. This species was previously reported in Algarve with an overall mean prevalence of 12% in canine populations in the mainland Portugal (e.g., prevalence rates of 16.7% in Ribatejo, 16.5% in Alentejo, 30% in Madeira 6.8% in Aveiro and 8.8% in Coimbra) (Alho *et al*., 2018). Despite the known endemicity of *D. immitis* in dogs from Portugal, *Cx. theileri* was the only species found to be infected with this filarioid (Ferreira *et al*., 2015). In addition, other mosquito species (e.g., *Culex pipiens*, Linnaeus, 1758; *Anopheles maculipennis* sensu lato, Meigen 1818; *A. atroparvus*, *Ae. caspius* and *Aedes detritus* sensu lato, Haliday, 1833) have been found positive for DNA of *D. immitis* in Portugal and thus have been suggested as potential vectors (Ferreira *et al*., 2017). Therefore, the molecular detection also of *D. repens* in *Cx. p. quinquefasciatus*, indicates that this mosquito species may be involved in the transmission of both *Dirofilaria* species. Though *D. repens* microfilariae (mfs) were diagnosed in a dog from the western part of Algarve region (Maia *et al*., 2016; Capelli *et al*., 2018), this filarioid has never been found in any mosquito collected in Portugal (Ferreira *et al*., 2015; Ferreira *et al*., 2017).

The same mosquito species, *Cx. p. quinquefasciatus*, as well as *Oc. caspius* was molecularly positive for *A. reconditum* (prevalence of infection 0.2 and 3%, respectively). Detection of *A. reconditum* DNA in both mosquito species above mentioned was unexpected as fleas and lice are the known vectors for this filarioid (Brianti *et al*., 2012). Accordingly, *A. reconditum* could have been acquired by mosquitoes while feeding on infected dogs, as suggested by the positivity of the same specimens for blood of dogs. Indeed, though xenomonitoring may be sensitive in detecting filarioids in insects, positive results for parasite DNA do not imply the role of them as vectors of the same pathogens, since larval stages can be detected up to 2 weeks after the meal with blood circulating mfs (Fischer *et al*., 2007). In addition, data above represent confirmatory evidence for the circulation of *A. reconditum* among canine populations from Portugal, with an estimated prevalence of the infection up to 0.8% (Ferreira *et al*., 2017).

The molecular detection in *Cx. p. quinquefasciatus* and *Cs. longiareolata* of unidentified filarioids, phylogenetically close to Onchocercidae and Filarioidea found in birds from Germany (Czajka *et al*., 2012) and Hungary (Kemenesi *et al*., 2015) could be due to the ornithophilic attitude of these mosquito species (Rizzoli *et al*., 2015). This hypothesis is also supported by the detection of common black bird (*Turdus merula*) DNA, in the blood meal of *Cs. longiareolata*, which suggests that this mosquito species may acquire mfs while feeding on birds.

On the other hand, the detection of human blood DNA in *Cx. p. quinquefasciatus* may be explained by the presence of two ecoforms within the *Cx. pipiens* complex, which occur in sympatry in Portugal (Gomes *et al*., 2009). Hence, the mosquito belonging to *Cx. pipiens* complex had a shift in the feeding preferences from ornithophilic (*pipiens*) to mammalophilic (*molestus*) and thus acquired the ability to feed on both hosts (Gomes *et al*., 2009). The plasticity of *Cx. p. quinquefasciatus* host preference has been previously described, indicating that this species of mosquito can act as a bridge vector for pathogens biting humans and other mammals and birds (Farajollahi *et al*., 2011).

Detection of the DNA of *O. lupi* in *Cx. p. quinquefasciatus* and *Cx. laticinctus* suggests the potential implication of mosquitoes belonging to genus *Culex* in the transmission of this filarioid. Though this result does not imply any evidence of their role as vectors for this filarial species, the recent findings of *Onchocerca* sp. larvae from the sand fly *Psychodopygus carrerai carrerai* (Brilhante *et al*., 2020) suggests that insect species, other than simuliids, may acquire mfs of this onchocercid. Though *Onchocerca* sp. are known to be vectored by *Simulium* sp. and *Culicoides* sp. through cutting or chewing mouth parts (telmophagy/pool feeding), it is demonstrated that other blood‐feeding insects (e.g., *Aedes aegypti* mosquito) may obtain blood also by lacerating vessels and not only directly from vessels (Gordon & Lumsden, 1939). Therefore, mosquitoes may acquire the pathogen while feeding, by piercing mouth parts (solenophagy/tube feeding) and ingest mfs of *O. lupi* in subcutaneous connective tissue. While the potential role of black flies in the transmission of *O. lupi* in dogs and humans has been hypothesized, no convincing scientific evidence in this regard has yet been produced (Otranto *et al*., 2013a). To date, vector tracking of this filarioid is limited to the detection of the DNA of *O. lupi* in *S. tribulatum* in the USA (Hassan *et al*., 2015) and to the presence of *Simulium reptans* in areas where the cases of canine ocular onchocerciasis have been reported (i.e., Switzerland, Germany and Hungary) (Otranto *et al*., 2013a).

Findings of *Wolbachia* supergroup B in *Cx. p. quinquefascaitus* confirms previous reports in this vector species (Werren *et al*., 2008), whereas the detection of *Wolbachia* supergroup E in *Cx. laticinctus* positive for *O. lupi* is new to science and it needs further investigation. In the same line of reason, implications of this *Wolbachia* supergroup in the vector capacity of this mosquito species needs further research. Indeed, it is known that *Wolbachia* symbionts are able to manipulate the reproductive system of many invertebrate hosts increasing or decreasing host fitness for the developing pathogens in them (Zug & Hammerstein, 2015). Furthermore, a mutualistic symbiosis has been described between *Wolbachia* and Onchocercidae, which contribute to the reproduction of filariae (Casiraghi *et al*., 2004).

## Conclusion

Detection of the DNA of *O. lupi* as well as of *D. repens* in *Culex* spp. for the first time in Portugal needs further investigations to understand their vector capacity and competence to transmit these zoonotic parasites to animals and humans in Portugal. Algarve is a touristic place offering a wide range of outdoor activities (ie., bird watching, fishery and aquaculture) that may facilitate the spread of filarioids vectored by dipteran species. Thus, information regarding the vectors and their transmitting pathogens may help to standardise an adequate prophylactic approach and proper vector control measures to effectively control the above zoonotic filarioids.

## Authors' contributions

Sample collection: Maria Alfonsa Cavalera; Carla Maia; Conceptualization: Ranju R.S. Manoj, Maria Stefania Latrofa, Domenico Otranto; Supervision: Maria Stefania Latrofa, Domenico Otranto; Methodology: Ranju R.S. Manoj, Maria Stefania Latrofa; Investigation: Ranju R.S. Manoj, Maria Stefania Latrofa; Maria Alfonsa Cavalera; Data curation: Ranju R.S. Manoj, Maria Stefania Latrofa; Writing ‐ original draft: Ranju R.S. Manoj, Maria Stefania Latrofa; Writing ‐ review & editing: Ranju R.S. Manoj, Maria Stefania Latrofa, Domenico Otranto, Maria Alfonsa Cavalera, Jairo Alfonso Mendoza‐Roldan, Carla Maia. All authors read and approved the final manuscript.

## Supporting information

**Appendix S1.** Primers used for the study

**Appendix S2.** Details of the blood meal analysis of positive flies

**Appendix S3.** Gene Bank accession numbers of the representative sequences of filarioids and *Wolbachia* identified in the current study

## Data Availability

Data obtained from the results of this manuscript are included within the article. The raw data set used and analysed during the current study are available from the corresponding author upon reasonable request.
