# Supplementary material for: Molecular detection of zoonotic filarioids in Culex spp. from Portugal
Source: Med Vet Entomol. 2021 May 4;35(3):468–77. doi: 10.1111/mve.12524 (PMC8453905; doi:10.1111/mve.12524)
Supplement: Supplementary file 1 — Appendix S1. Primers used for the study [file MVE-35-468-s001.doc]

**Appendix 1. Primers used for the study**

| **Targets** | **Target gene** | **Primer sequence** | **Reference** |
| --- | --- | --- | --- |
| Filarioids | *cox*1 | NTF: 5’-TGATTGGTGGTTTTGGTAA-3’ | Otranto *et al*., 2011 |
|  |  | NTR: 5’-ATAAGTACGAGTATCAATATC-3’ |  |
| Filarioids | 12S rRNA | Fila_12SF2 (A): 5’-CGGGAGTAAAGTTTTGTTTAAACCG-3’ | Casiraghi *et al*., 2004 |
|  |  | Fila_12SR1new (C): 5’- CATTGACGGATGGTTTGTACCAC-3’ |  |
| Filarioids qPCR | *cox*1 | *O*.*l*.F 5’-GGAGGTGGTCCTGGTAGTAG-3’ | Latrofa *et al*., 2018 |
|  |  | *O*.*l*.R 5’-GCAAACCCAAAACTATA  GTATCC-3’ |  |
|  |  | Probe: FAM-5’-CTTAGAGTAGAGGGTCAG  CC-3’ |  |
| Mosquito identification | *cox*1 | MTFN: 5’-GGATTTGGAAATTGATTAGTTCCTT-3’ | Kumar *et al*., 2007 |
|  |  | MTRN: 5’-AAAAATTTTAATTCCAGTTGGAACAGC-3’ |  |
| Mosquito identification | *cox*1 | UEA7: 5’-TACAGTTGGAATAGACGTTGATAC -3’ | Otranto *et al*., 2003 |
|  |  | UEA10: 5’-AAAAATGTTGAGGGAAAAATGTTA-3’ |  |
| Blood meal | *cytochrome b* | Cyto1: 5’-CCA TCA AAC ATC TCA GCA TGA TGA AA-3’ | Abbasi *et al*., 2009 |
|  |  | Cyto2: 5’-CCC CTC AGA ATG ATA TTT GTC CTC-3’ |  |
| *Wolbachia* | 16S rRNA | EHR-16SD: 5’-GGTACCYACAGAAGAAGTCC-3’ | Martin *et al*. 2005 |
|  |  | EHR-16SR: 5’-TAGCACTCATCGTTTACAGC-3’ |  |
| *Wolbachia* | *wsp* | WSPF: 5’-TGGTCCAATAAGTGATGAAGAAAC-3’ | Zou *et al*., 1998 |
|  |  | WSPR: 5’-AAAAATTAAACGCTACTCCA-3’ |  |
