# Supplementary material for: Molecular detection of zoonotic filarioids in Culex spp. from Portugal
Source: Med Vet Entomol. 2021 May 4;35(3):468–77. doi: 10.1111/mve.12524 (PMC8453905; doi:10.1111/mve.12524)
Supplement: Supplementary file 2 — Appendix S2. Details of the blood meal analysis of positive flies [file MVE-35-468-s002.doc]

**Appendix 2: Details of the blood meal analysis of positive flies**

| **Sl. No.** | **Mosquito species** | **Pathogen** | **Host blood detected** |
| --- | --- | --- | --- |
| 1. | *Culex pipiens quinquefasciatus* | *D.immitis* | *Homo sapiens* |
| 2. | *Culiseta longiareolata* | Onchocercidae sp. | *Turdus merula* |
| 3 | *Ochlerotatus caspius* | *Acanthocheilonema reconditum* | *Canis lupus familiaris* |
| 4. | *Culex pipiens quinquefasciatus* | Onchocercidae sp. | *Homo sapiens* |
| 5 | *Ochlerotatus caspius* | *Acanthocheilonema reconditum* | *Homo sapiens* |
| 6 | *Culiseta longiareolata* | Onchocercidae sp. | *Turdus merula* |
