# Supplementary material for: Molecular detection of zoonotic filarioids in Culex spp. from Portugal
Source: Med Vet Entomol. 2021 May 4;35(3):468–77. doi: 10.1111/mve.12524 (PMC8453905; doi:10.1111/mve.12524)
Supplement: Supplementary file 3 — Appendix S3. Gene Bank accession numbers of the representative sequences of filarioids and Wolbachia identified in the current study [file MVE-35-468-s003.doc]

**Appendix 3. Gene Bank accession numbers of the representative sequences of filarioids and *Wolbachia* identified in the current study**

| **Organism** | **Host** | **Gene** | **Accession number** |
| --- | --- | --- | --- |
| *Wolbachia pipientis* | *Culex quinquefasciatus* | 16S rRNA | MW242746 |
| *Wolbachia pipientis* | *Culex latcinctus* | 16S rRNA | MW242747 |
| *Wolbachia pipientis* | *Ochlerotatus caspius* | 16S rRNA | MW242748 |
| *Culex     laticinctus* | - | *cox* 1 | MW243588 |
| *Ochlerotatus caspius* | - | *cox*1 | MW243595 |
| *Ochlerotatus caspius* | - | *cox*1 | MW243596 |
| *Culex pipiens quinquefasciatus* | - | *cox*1 | MW243597 |
| *Culiseta longiareolata* | - | *cox*1 | MW243598 |
| *Acanthocheilonema reconditum* | *Culex pipiens quinquefasciatus* | *cox*1 | MW246127 |
| *Dirofilaria repens* | *Culex pipiens quinquefasciatus* | *cox*1 | MW246128 |
| *Dirofilaria immitis* | *Culex pipiens quinquefasciatus* | *cox*1 | MW246129 |
| Filaroidea sp. | *Culiseta* *longiareolata* | *cox*1 | MW246130 |
| Filaroidea sp. | *Culex pipiens quinquefasciatus* | *cox*1 | MW246131 |
| *Onchocerca lupi* | *Culex pipiens quinquefasciatus, Culex laticinctus* | *cox*1 | MW246132 |
| *Onchocerca_lupi* | *Culex pipiens quinquefasciatus, Culex laticinctus* | 12S rRNA | MW254894 |
| *Acanthocheilonema_reconditum* | *Oclherotatus caspius* | 12S rRNA | MW254895 |
| *Dirofilaria repens* | *Culex pipiens quinquefasciatus* | 12S rRNA | MW254896 |
| *Dirofilaria immitis* (6) | *Culex pipiens quinquefasciatus* | 12S rRNA | MW254897 |
| *Dirofilaria immitis* (1) | *Culex pipiens quinquefasciatus* | 12S rRNA | MW254898 |
| Onchocercidae_3 | *Culex pipiens quinquefasciatus, Culiseta longiareolata* | 12S rRNA | MW254899 |
| Onchocercidae_2 | *Culex pipiens quinquefasciatus, Culiseta longiareolata* | 12S rRNA | MW254900 |
| Filaroidea | *Culex pipiens quinquefasciatus* | 12S rRNA | MW254901 |
| *Wolbachia pipientis* | *Culex pipiens quinquefasciatus* | *wsp* | MW435608 |
| *Wolbachia pipientis* | *Culex laticinctus* | *wsp* | MW435609 |
| *Wolbachia pipientis* | *Culex pipiens quinquefasciatus* | *wsp* | MW435610 |
| *Homo sapiens* | *Culex pipiens quinquefasciatus* | *Cytochrome* b | MW435611 |
| *Homo sapiens* | *Oclherotatus caspius* | *Cytochrome* b | MW435612 |
| *Turdus merula* | *Culiseta longiareolata* | *Cytochrome* b | MW435613 |
| *Canis lupus familiaris* | *Oclherotatus caspius* | *Cytochrome* b | MW435614 |
